# Erythromycin Enhances CD4^+^Foxp3^+^ Regulatory T-Cell Responses in a Rat Model of Smoke-Induced Lung Inflammation

**DOI:** 10.1155/2012/410232

**Published:** 2012-05-31

**Authors:** Jing Bai, Shi-Lin Qiu, Xiao-Ning Zhong, Qiu-Ping Huang, Zhi-Yi He, Jian-Quan Zhang, Guang-Nan Liu, Mei-Hua Li, Jing-Min Deng

**Affiliations:** Department of Respiratory Medicine, The First Affiliated Hospital of Guangxi Medical University, Nanning 530000, China

## Abstract

Heavy smoking can induce airway inflammation and emphysema. Macrolides can modulate inflammation and effector T-cell response in the lungs. However, there is no information on whether erythromycin can modulate regulatory T-cell (Treg) response. This study is aimed at examining the impact of erythromycin on Treg response in the lungs in a rat model of smoking-induced emphysema. Male Wistar rats were exposed to normal air or cigarette smoking daily for 12 weeks and treated by gavage with 100 mg/kg of erythromycin or saline daily beginning at the forth week for nine weeks. The lung inflammation and the numbers of inflammatory infiltrates in bronchoalveolar lavage fluid (BALF) were characterized. The frequency, the number of Tregs, and the levels of Foxp3 expression in the lungs and IL-8, IL-35, and TNF-*α* in BALF were determined by flow cytometry, RT-PCR and ELISA, respectively. Treatment with erythromycin reduced smoking-induced inflammatory infiltrates, the levels of IL-8 and TNF-*α* in the BALF and lung damages but increased the numbers of CD4^+^Foxp3^+^ Tregs and the levels of Foxp3 transcription in the lungs, accompanied by increased levels of IL-35 in the BALF of rats. Our novel data indicated that erythromycin enhanced Treg responses, associated with the inhibition of smoking-induced inflammation in the lungs of rats.

## 1. Introduction

Chronic obstructive pulmonary disease (COPD) is one of the most prevalent illnesses worldwide and is estimated as the third leading cause of mortality in 2020 [1]. COPD is characterised by airflow limitation that is poorly reversible. The pathogenesis of COPD is usually progressive and associated with an abnormal inflammatory response in the lungs, particularly in response to noxious particles or gases, such as cigarette smoke [2]. Recently, COPD-associated inflammation is thought to be an autoimmune response induced by smoking or pathogenic microbials that activate lymphocytes and antigen-presenting cells [3]. Previous studies have shown that Th1 cells are predominantly associated with the development of emphysematous lungs, leading to the progression of COPD although the mechanisms by which tobacco smoke is associated with Th1 immunity remain unclear [4–7].

CD4^+^CD25^+^Foxp3^+^ regulatory T cells (Tregs) are crucial regulators of the maintenance of peripheral immunologic tolerance, and Tregs can suppress effectors Th1, Th2, and Th17 responses, inflammation, and autoimmune responses [8, 9]. Tregs can secrete IL-35, which inhibits inflammatory responses [10]. A deficiency in Treg regulation has been associated with the development of many Th1-mediated chronic inflammation and autoimmune disorders, including type 1 diabetes, multiple sclerosis, atherosclerosis, and rheumatoid arthritis [11–14]. Interestingly, decreased numbers of Tregs were detected in the lungs of subjects with emphysema [15], suggesting that Tregs participate in the regulation of emphysema-related inflammation in the lungs. However, little is known on what therapeutic strategies could increase the number of Tregs and IL-35 responses in the lungs of subjects with emphysema-related inflammation. Currently, anti-inflammatory steroids have been often used for the treatment of COPD patients with acute exacerbation, but the therapeutic efficacy of steroids is limited [16, 17]. Therefore, discovery of new therapeutic reagents will be of great significance in the management of patients with COPD.

Erythromycin is a 14-membered ring macrolide antibiotic and has been prescribed for the treatment of various respiratory infections. Erythromycin can inhibit mitogen-stimulated human T-cell proliferation and cytokine production, which are associated with inhibition of the MAPK and NF-*κ*B activation [18, 19]. Furthermore, erythromycin can ameliorate chronic inflammation in various animal models [20, 21]. In addition, long-term treatment with low doses of a 14-membered ring macrolide is beneficial for patients with airway inflammatory diseases, such as diffuse panbronchiolitis (DPB) [22], cystic fibrosis [23, 24], bronchiectasis [25], and bronchial asthma [26, 27]. Our previous study has reported that treatment with erythromycin reduces the number of smoking-induced airway inflammatory infiltrates and airway remodelling in the lungs of rodents [28]. However, little is known on whether treatment with erythromycin could modulate Treg and IL-35 responses in the lungs.

In this study, we evaluated the impact of treatment with erythromycin for nine weeks on cigarette smoking-induced inflammation in a rat model of emphysema. Our findings indicated that treatment with erythromycin not only reduced smoking-induced airway inflammation and emphysema but also increased Treg infiltrates and IL-35 production in the lungs of rats.

## 2. Materials and Methods

### 2.1. Animals and Treatments

Male Wistar rats at 12 weeks of age were obtained from the Animal Research Center of Guangxi Medical University. The animals were housed individually in standard laboratory cages with free access to standard food and tap water *ad libitum*. The experimental protocols were established, according to the guidelines of NIH Animal Research Care and were approved by the Animal Research Care Committee of Guangxi Medical University.

Individual rats (*n* = 40) were exposed either to room air (control) or to cigarette smoke, as described previously [28]. Briefly, groups of rats (*n* = 20 per group) were exposed to tobacco smoke with 20 cigarettes (Nanning Jiatianxia unfiltered cigarettes: 12 mg of tar and 0.9 mg of nicotine) in a closed 0.54 m^3^ space for 2 hours daily for six consecutive days per week for 12 consecutive weeks. As a result, an optimal ratio of smoking to air at 1 : 6 was obtained and the levels of oxygen exposed by the rats were kept at a 21 ± 1%, which is similar to atmospheric oxygen concentrations. The rats tolerated the cigarette smoke without evidence of toxicity (the levels of serum carboxyhemoglobin in rats were at ~10%, and no weight loss in the rats was observed). The levels of serum carboxyhemoglobin in the smoking rats (*n* = 20) were 8.3 ± 1.4%, as compared with 1.0 ± 0.2% in the control rats (*n* = 20), which were similar to the concentrations of blood carboxyhemoglobin of human smokers [29].

Three weeks after exposure to cigarette smoke, the rats were randomized and treated by gavage with 100 mg/kg/d of erythromycin (Meichuang Pharmaceuticals, Dailian, China) in saline (1 mL) or saline alone daily for nine weeks, respectively. We used this dose based on our previous findings to show that treatment with 100 mg/kg/d of erythromycin inhibits smoke-related lung inflammation without obvious adverse effect [28]. The rats that exposed to regular air were randomized and treated with erythromycin or saline in the same manner. Accordingly, there were four groups of rats (*n* = 10 per group). The normal group of rats were exposed to regular air and treated with saline (group N); the smoking group of rats were exposed to smoking air for 12 weeks and treated with saline (group S); the erythromycin group of rats were exposed to smoking air for 12 weeks and treated with erythromycin (group E); the control group of rats were exposed to regular air and treated with erythromycin (group C).

One day after the last smoking, animals were injected intraperitoneally with 20 mg/kg pentobarbital and subjected to a thoracotomy. Their left lungs were lavaged through an intratracheal cannula three times with 2 mL of cold saline, and the bronchoalveolar lavage fluid (BALF) samples were collected. The left lungs were used for the preparation of single cell suspension. The lower lobes of their right lungs were fixed in 10% formalin for pathological examination.

### 2.2. Histology

The fixed lower lobes of the right lungs were embedded in paraffin, and the midsagittal sections of the lungs were stained with hematoxylin and eosin (H&E), followed by examining under a light microscope. Three non-consecutive lung sections from each animal and three non-overlapping random fields from each section were examined for the quantification of lung damages. Alveolar airspace enlargement was assessed by the mean linear intercept (MLI) by two independent individuals in a blinded manner, as described previously [30]. Briefly, multiple digital images of histological sections were systematically captured at 100 × magnification. Images were overlaid with a 10 × 10 grid (1 mm^2^), and the MLI was established from every second image (i.e., in a checkerboard fashion, averaging six images for each rat). The distribution of the MLI values of all the digital photographs was assessed using frequency distribution analysis and characterized using a Gaussian model.

### 2.3. Characterization of Inflammatory Cells in BALF

The collected BALF samples from the left lung tissues were centrifuged, and their supernatants were stored at −80°C for ELISA analysis. The pelleted cells were resuspended in PBS and a portion of the cells (1 × 10^5^ cells) was subjected to cytospin centrifugation on glass slides and fixed with methanol, followed by staining with May-Grünwald-Giemsa solution, and a differential cell count was performed under a light microscope, according to morphological characteristics.

### 2.4. Measurement of IL-8, IL-35, and TNF-*α* in BALF

The concentrations of IL-8, IL-35, and TNF-*α* in BALF were measured with a multiplex-enzyme-linked immunosorbent assay (ELISA) system, according to the manufacturers' instructions (Lincoplex Systems, St Charles, MO, USA).

### 2.5. Lung Cell Preparation

A single-cell suspension of whole left lung tissue was prepared by combined procedures of mechanical fragmentation, enzymatic digestion, and centrifugation, as described in previous studies [5, 15]. The prepared lung cells were used for flow cytometry analyses. Briefly, lungs were flushed via the right ventricle with 10 mL of warm (37°C) HBSS (calcium and magnesium free) containing 5% fetal bovine serum (FBS, Sigma, Beijing, China), 100 U/mL of penicillin, and 100 *μ*g/mL of streptomycin (Gibco BRL). The lungs were then cut into small pieces (~2 mm in diameter) and digested with 150 U/mL of collagenase (Worthington Biochemical, Freehold, NJ, USA) in HBSS with being shaken at 37°C for 1 h. Using a plunger from a 5-mL syringe, the lung pieces were triturated through a mess of 100 *μ*M into HBSS, and the resulting cell suspension was filtered through nylon mesh. The cells were washed twice, and mononuclear cells were isolated using density centrifugation in 30% percoll (Pharmacia, Uppsala, Sweden). The total numbers of cells were counted. The collected leukocytes (1 × 10^6^ cells) were used for flow cytometry analysis and the remaining cells were used for the extraction of total RNA for RT-PCR analysis.

### 2.6. Flow Cytometry

The collected cells (1 × 10^6^) from individual rats were stained with PE-Cy5-conjugated anti-CD4 (clone: OX-35) or its isotype control (BD Pharmingen, San Diego, CA, USA) at 4°C for 45 minutes, fixed, permeabilized, and stained with PE-conjugated anti-Foxp3 (clone: FJK16s) or its isotype control (eBioscience, Wembley, UK) at 4°C for another 40 minutes. The frequency and the number of Tregs were determined by flow cytometry on a FACSCalibur (BD PharMingen) and analysed by FCS Express software.

### 2.7. RNA Isolation and RT-PCR

Total RNA was extracted from the lung cells of individual rats with TRIzol reagent, according to the manufacturers' instructions (Invitrogen, Carlsbad, CA, USA). The quality and quantity of total RNA were analysed by a spectrophotometer. The RNA samples were reversely transcribed into cDNA using a reverse transcription kit (Finn-zymes, Espoo, Finland) and oligo (dT) primers. The relative levels of Foxp3 mRNA transcripts to control *β*-actin in individual samples were characterized by quantitative RT-PCR using SYBR Green on a LightCycler (iCycler IQ, BioRad, USA) and the specific primers. The sequences of primers were forward 5′-GGAGATTACTGCCCTGGCTCCTA-3′, and reverse 5′-GACTCATCGTACTCCTGCTTGCTG-3′ for *β*-actin and forward 5′-TGAGCTGGCTGCAATTCTGG-3′ and reverse 5′-ATCTAGCTGCTCTGCATGAGGTGA-3′ for Foxp3. The PCR amplifications were performed in triplicate at 95°C for 30 sec and subjected to 40 cycles of 95°C for 5 sec and 60°C for 30 sec. The values of Foxp3 mRNA transcripts in each sample were normalized to that of *β*-actin and the relative levels of Foxp3 mRNA transcripts were calculated.

### 2.8. Statistical Analysis

Data are expressed as means ± SD. Differences among groups were analysed using the analysis of variance (ANOVA) and post hoc Student's *t*-test, the Kruskal-Wallis test, and the Mann-Whitney *U*-test where applicable using statistical package SPSS 11.0 (SPSS, Chicago, IL, USA). The association between two variants was analyzed using Spearman's rank method. A *P* value of <0.05 was considered statistically significant.

## 3. Results

### 3.1. Treatment with Erythromycin Reduces the Smoking-Induced Lung Damages in Rats

**Figure 1:**
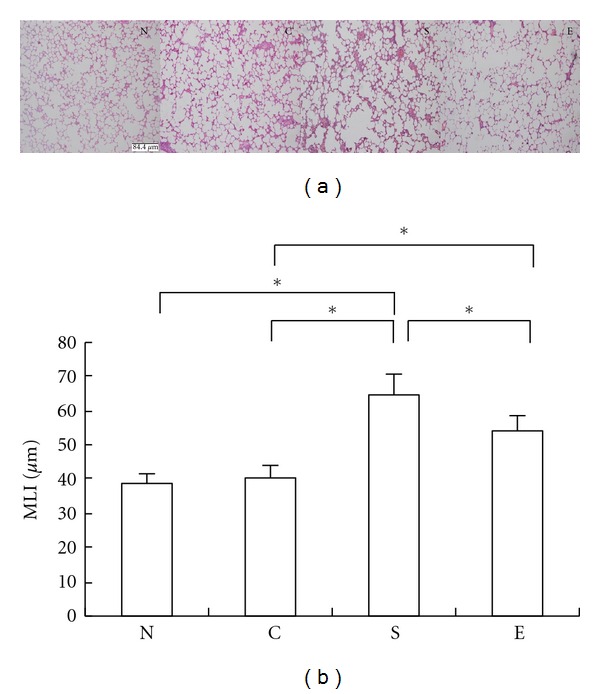
Treatment with erythromycin protects against the smoking-induced emphysema in rats. The lung tissue sections from different groups of rats were subjected to H&E staining, and the alveolar airspace enlargement was assessed using MLI by two independent individuals in a blinded manner. Data are representative images or expressed as mean value ± SD of each group of rats (*n* = 10) from five separate experiments. (a) Morphological changes in the lungs of rats (magnification × 100). (b) Quantitative analysis of alveolar airspace. Group N: rats exposed to regular air without any special treatment; Group C: rats exposed to regular air and were treated with erythromycin; Group S: rats exposed to smoking air and were treated with saline; Group E: rats exposed to smoking air and were treated with erythromycin daily for nine weeks beginning at the forth weeks smoking. *P < 0.05.

Following smoking for 12 weeks and treatment with erythromycin for 9 weeks, the lung tissue sections of the different groups of rats were stained with H&E and subjected to quantitative analysis of the lung airspace (Figure 1). We observed the enlargement of air spaces and many inflammatory infiltrates in the lungs of the smoking rats. Quantitative analysis indicated that there was no significant difference in the MLI values between the N and C groups of rats. In contrast, the MLI values in the S and E group of rats were significantly greater than that in the N and C groups of rats (*P* < 0.05), demonstrating that long-term heavy smoking-induced lung emphysema in rats. Interestingly, the MLI values in the E groups of rats were significantly less than that in the S group of rats although they remained greater than that in controls. In addition, treatment with erythromycin mitigated smoke-induced histological damage in the lungs of rats, consistent with our previous observation [28]. These data indicated that treatment with erythromycin significantly diminished smoking-related emphysema in the lungs of rats.

### 3.2. Treatment with Erythromycin Modulates the Smoking-Induced Inflammatory Infiltrates in BALF

**Figure 2:**
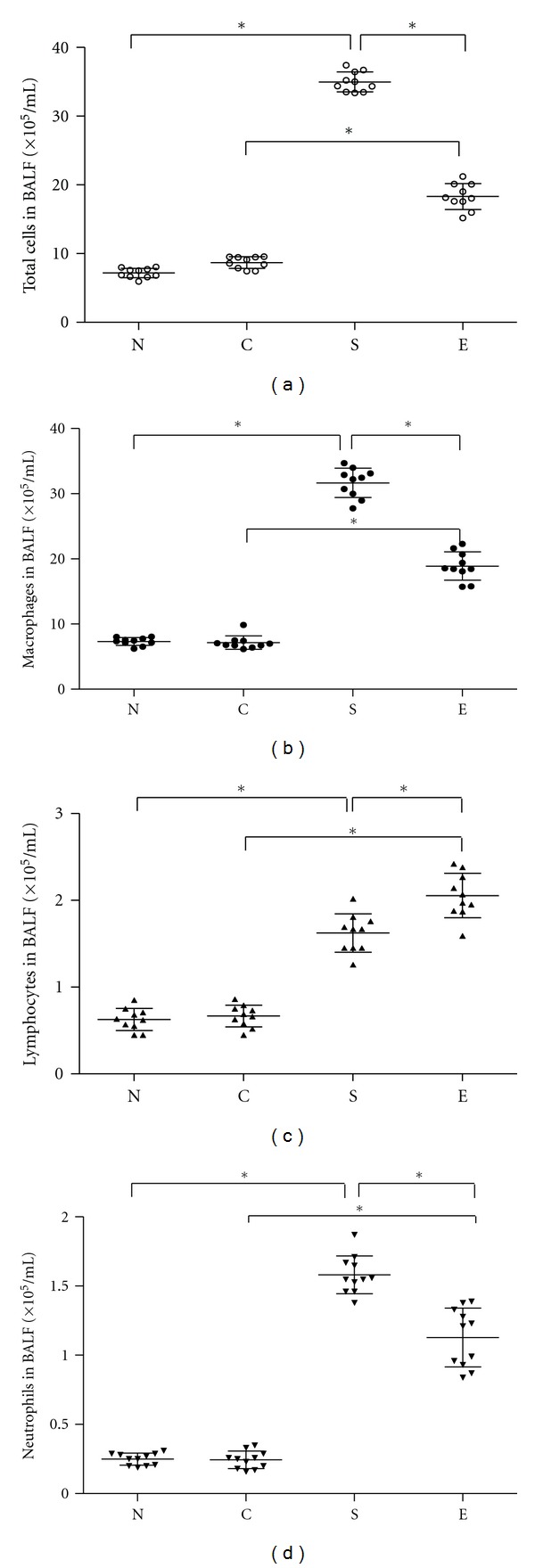
Treatment with erythromycin reduces the numbers of inflammatory infiltrates in the lungs of rats. BALF samples were collected from individual rats and the cells were stained with May-Grünwald-Giemsa. The numbers of total inflammatory infiltrates, macrophages, lymphocytes, and neutrophils were analyzed, according to their morphological characters. Data are expressed as mean numbers of individual samples and mean values (lines) for each group (*n* = 10). Group N: rats exposed to regular air without any special treatment; Group C: rats exposed to regular air and were treated with erythromycin; Group S: rats exposed to smoking air and were treated with saline; Group E: rats exposed to smoking air and were treated with erythromycin daily for nine weeks beginning at the forth weeks smoking. *P < 0.05.

To quantify the airway inflammation response, we evaluated the numbers of inflammatory infiltrates in BALF and found significantly increased numbers of total infiltrates, particularly macrophages, lymphocytes, and neutrophils in the BALF from the smoking rats, as compared with that in the N and C groups of rats (*P* < 0.05, Figure 2). In contrast, the total numbers of inflammatory infiltrates, macrophages, lymphocytes, and neutrophils in the BALF from the erythromycin-treated smoking rats were reduced significantly, as compared with those in the smoking rats without erythromycin treatment. In addition, treatment with erythromycin did not cause obvious adverse effect in rats, consistent with our previous findings [28]. These data demonstrated that treatment with erythromycin significantly mitigated smoking-induced inflammatory cell infiltration in the lungs of rats.

### 3.3. Treatment with Erythromycin Alters the Levels of TNF-*α* and IL-8 in BALF

**Figure 3:**
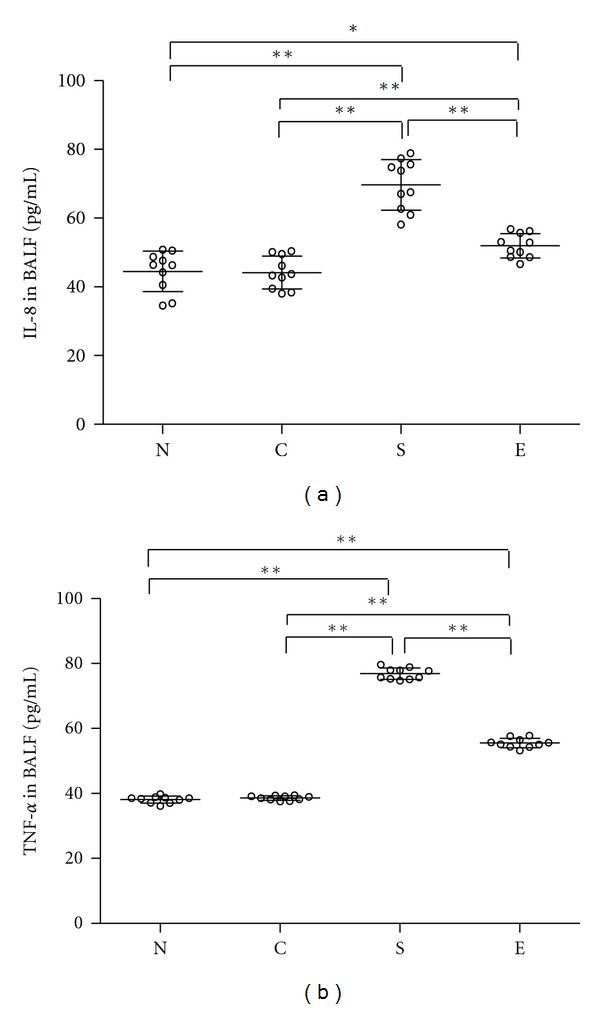
Treatment with erythromycin decreases the levels of TNF-*α*, IL-8 in the lungs of rats. The levels of TNF-*α* and IL-8 in BALF of individual rats were analyzed by ELISA. Data shown are mean values of individual samples from three separate experiments and mean values for each group of rats (*n* = 10). Group N: rats exposed to regular air without any special treatment; Group C: rats exposed to regular air and were treated with erythromycin; Group S: rats exposed to smoking air and were treated with saline; and Group E: rats exposed to smoking air and were treated with erythromycin daily for nine weeks beginning at the forth weeks smoking. *P < 0.05, **P < 0.01.

Analysis of the concentrations of TNF-*α* and IL-8 in the BALF indicated that significantly higher levels of TNF-*α* and IL-8 were detected in BLAF from the smoking rats, as compared with that in the N and C groups of rats (Figure 3). Furthermore, the levels of TNF-*α* and IL-8 in BALF from the smoking rats that had been treated with erythromycin were significantly lower than that in the smoking rats without erythromycin treatment. Apparently, treatment with erythromycin inhibited the smoking-induced proinflammatory cytokine production in the lungs.

### 3.4. Treatment with Erythromycin Alters the Numbers of Tregs in the Lungs of Rats

**Figure 4:**
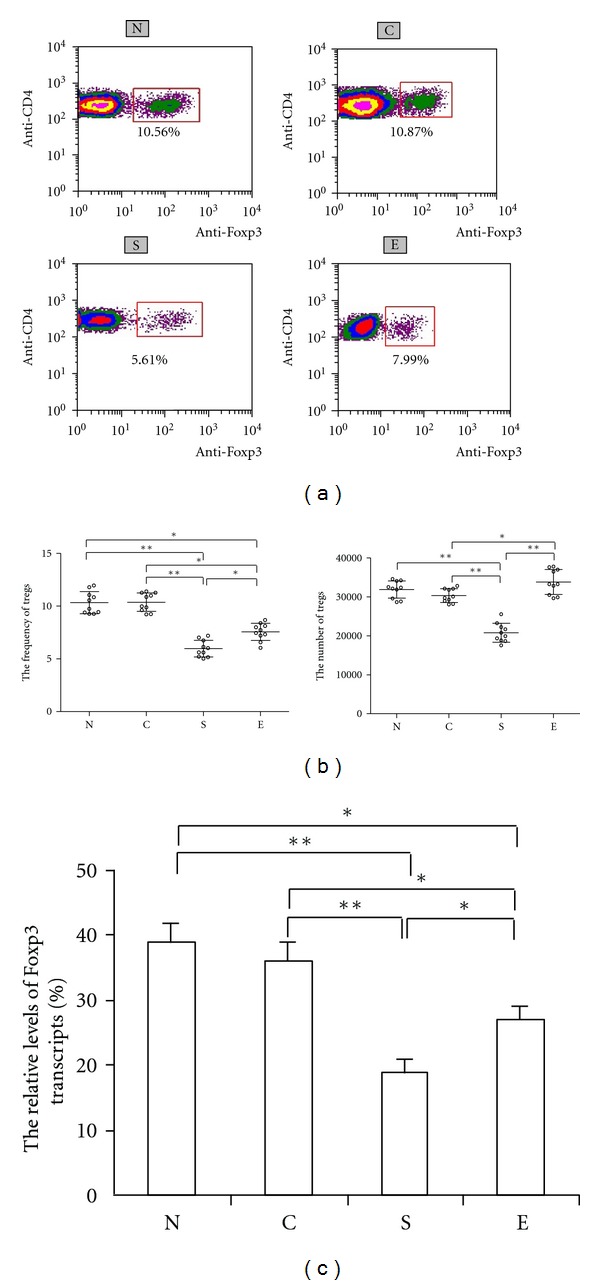
Treatment with erythromycin modulates the frequency and the number of Treg and Foxp3 transcription in the lungs of rats. The frequency of Tregs, the number of Tregs (b), and the relative levels of Foxp3 mRNA transcripts to *β*-actin in the lungs (c) were analyzed by flow cytometry (a) and RT-PCR, respectively. The isolated lung cells were stained with anti-CD4 and anti-Foxp3 and subjected to flow cytometry analysis. Data are expressed as mean numbers of individual samples and mean values (lines) of each group or the mean ± SD of the relative levels of Foxp3 mRNA transcripts of each group (*n* = 10 per group) of rats from three separate experiments. Group N: rats exposed to regular air without any special treatment; Group C: rats exposed to regular air and were treated with erythromycin; Group S: rats exposed to smoking air and were treated with saline; and Group E: rats exposed to smoking air and were treated with erythromycin daily for nine weeks beginning at the forth weeks smoking. *P < 0.05, **P < 0.01.

Flow cytometry analysis revealed that the frequency and the number of Tregs in the lung parenchyma of smoking rats were significantly lower than that of the N and C groups of control rats (*P* < 0.01, Figure 4), while the frequency and the number of Tregs in the erythromycin-treated group of rats were higher than that of the S group of rats (*P* < 0.05). A similar pattern of the relative levels of Foxp3 mRNA transcripts was detected in the different groups of rats. Apparently, treatment with erythromycin mitigated heavy smoking-induced reduction in the numbers of Tregs in the lungs of rats.

### 3.5. Treatment with Erythromycin Alters the Levels of IL-35 in BALF

**Figure 5:**
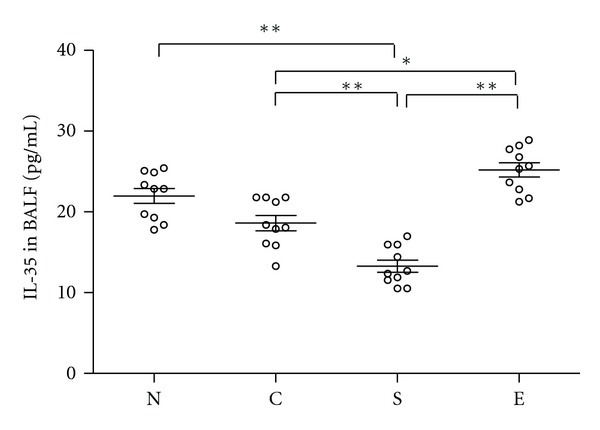
Treatment with erythromycin increases the levels of IL-35 in the lungs of rats. The levels of IL-35 in BALF of individual rats were analyzed by ELISA. Data shown are mean values of individual samples from three separate experiments and mean values (lines) of each group of rats (*n* = 10). Group N: rats exposed to regular air without any special treatment; Group C: rats exposed to regular air and were treated with erythromycin; Group S: rats exposed to smoking air and were treated with saline; and Group E: rats exposed to smoking air and were treated with erythromycin daily for nine weeks beginning at the forth weeks smoking. *P < 0.05, **P < 0.01.

IL-35 is an inhibitory cytokine and is predominantly secreted by Tregs. Next, we determined the levels of IL-35 in BALF from different groups of rats. The concentrations of IL-35 in the BALF from the S group of rats were significantly lower than that in the N and C groups of control rats (Figure 5). Interestingly, the levels of IL-35 in the BALF from E group of rats were similar to that in the N and C groups of rats and were significantly higher than that in the S group of rats. Apparently, treatment with erythromycin increased the levels of IL-35 responses in the lungs of rats.

## 4. Discussion

COPD and emphysema are common destructive inflammatory diseases that are leading causes of mortality worldwide. The smoking-induced emphysema is thought to be an autoimmune disease and is mediated predominantly by Th1 responses in the lung [15]. In this study, we employed a rat model of smoking-related airway inflammation and emphysema to test the therapeutic effect of treatment with erythromycin and the potential mechanisms. Our data showed that treatment with erythromycin significantly reduced smoking-induced lung inflammation and damages, consistent with our previous findings [28]. Furthermore, treatment with erythromycin increased the numbers of Tregs, accompanied by increased levels of inhibitory IL-35 in the lungs of rats. The increased levels of IL-35 may contribute to the inhibition of erythromycin on smoking-related inflammation. Our novel findings extend previous observations and suggest that erythromycin may be valuable for the intervention of airway inflammation by upregulating Treg responses in patients with COPD in the clinic.

Macrolide antibiotics have been used for the treatment of lung inflammation in patients with COPD in the clinic [31]. Previous studies have shown that macrolides, especially for erythromycin, can modulate immune responses and inhibit inflammation in patients with DB and CF [32]. Indeed, long-term treatment with a low dose of macrolide benefits patients with COPD by its anti-inflammatory activities. In this study, we employed a well-known cigarette-smoking-inuced rat emphysema model and examined the effect of treatment with erythromycin on the airway inflammation and lung damages. We detected high values of MLI, great numbers of inflammatory infiltrates, and high levels of TNF-*α* and IL-8 in the lungs of smoking rats, demonstrating that heavy smoking-inuced emphysema and airway inflammation in the lungs of rats. Furthermore, we found that treatment with erythromycin mitigated the smoking-induced emphysema and reduced the numbers of inflammatory infiltrates and levels of TNF-*α* and IL-8 in the lungs of rats. Our data were consistent with a previous report that treatment with clarithromycin for six months decreases airspace enlargement in the smoke-induced emphysema in mice [33]. Our findings support the notion that erythromycin inhibits airway inflammation [28].

Heavy smoking can modulate the function of antigen-presenting cells, which may induce T-cell autoimmunity against the lungs and Th1 immunity has been thought to be related to the pathogenic process of COPD [15, 34]. Microbials, such as erythromycin, can modulate T-cell responses and inhibit airway inflammation [23, 27, 35]. Notably, Tregs are potent regulators of T-cell autoimmunity and inflammation and IL-35 is predominantly produced by Tregs and contributes to regulatory T-cell function [8, 9]. We found that treatment with erythromycin enhanced Treg responses, which may contribute to the inhibition of airway inflammation. Evidentially, in comparison with that in the smoking rats, treatment with erythromycin significantly increased the frequency and the numbers of Treg infiltrates in the lungs. Furthermore, treatment with erythromycin upregulated the levels of Foxp3 mRNA transcripts in the lungs. In addition, treatment with erythromycin increased the levels of IL-35 in the BALF, given that Tregs can inhibit pathogenic T-cell responses and IL-35 is crucial for the function of Tregs [10]. Although the increased Treg responses in the lungs by treatment with erythromycin were moderate the significantly reduced inflammation suggests that marginal effect of erythromycin on increasing Treg response in the lung may be sufficient in suppressing smoking-related inflammation. We understand that our data did not demonstrate that the increased Treg responses were responsible for the inhibition of smoke-related lung inflammation. We are interested in further investigation of whether adoptive transfer of Tregs or inactivation of Tregs could modulate smoke-induced inflammation and examining whether neutralization of IL-35 could change the effect of treatment with erythromycin on smoke-induced lung damage in rats.

While there is clear evidence that treatment with macrolide antibiotics inhibits effector T-cell proliferation and cytokine production there currently is little information on how macrolide antibiotics modulate T-cell immunity. Erythromycin may modulate the components of gut microbiota and promote the development of Tregs. Indeed, the components of gut microbiota are crucial for the development of Tregs in rodents. Furthermore, a previous study has shown that Roxithromycin inhibits chemokine-induced chemotaxis of Th1 and Th2 cells but does not affect regulatory T-cell migration [36]. Erythromycin may act, like Roxithromycin, and inhibit the migration of effector T cells, but not Tregs, leading to relative increase in the numbers of Tregs in the lungs of rats. In addition, erythromycin has been shown to downregulate dendritic cell function and cytokine production, particularly for LPS-stimulated dendritic cell maturation and activation [37]. However, treatment with erythromycin does not affect peptidoglycan-induced dendritic cell activation [37]. It is possible that erythromycin may modulate dendritic cell function toward to promoting Treg development. Indeed, we found that treatment with erythromycin upregulated Foxp3 transcription and IL-35 production. Given that IL-35 has been shown to promote Treg proliferation the increased levels of IL-35 may feedback enhance Treg responses in the lungs of rats. We are interested in further investigating the mechanisms underlying the role of erythromycin in regulating Treg responses. 

## 5. Conclusions

In summary, treatment of COPD currently remains a significant challenge, and pharmacological understanding of drugs for the treatment of COPD is crucial for the control of disease progression. Our data indicated that treatment with erythromycin significantly reduced smoking-related lung inflammation and damages and modulated Treg and IL-35 responses in the lungs of rats. Therefore, our findings may provide new insights into understanding the pharmacological action of erythromycin in the management of COPD in the clinic.

## References

[1] Pauwels RA, Buist AS, Calverley PMA, Jenkins CR, Hurd SS (2001). Global strategy for the diagnosis, management, and prevention of chronic obstructive pulmonary disease: NHLBI/WHO Global Initiative for Chronic Obstructive Lung Disease (GOLD) workshop summary. *American Journal of Respiratory and Critical Care Medicine*.

[2] Rabe KF, Hurd S, Anzueto A (2007). Global strategy for the diagnosis, management, and prevention of chronic obstructive pulmonary disease: GOLD executive summary. *American Journal of Respiratory and Critical Care Medicine*.

[3] Agustí A, MacNee W, Donaldson K, Cosio M (2003). Hypothesis: does COPD have an autoimmune component?. *Thorax*.

[4] Saetta M, Mariani M, Panina-Bordignon P (2002). Increased expression of the chemokine receptor CXCR3 and its ligand CXCL10 in peripheral airways of smokers with chronic obstructive pulmonary disease. *American Journal of Respiratory and Critical Care Medicine*.

[5] Grumelli S, Corry DB, Song LZ (2004). An immune basis for lung parenchymal destruction in chronic obstructive pulmonary disease and emphysema. *PLoS Medicine*.

[6] Cosio MG, Guerassimov A (1999). Chronic obstructive pulmonary disease: inflammation of small airways and lung parenchyma. *American Journal of Respiratory and Critical Care Medicine*.

[7] Shevach EM (2000). Regulatory T cells in autoimmmunity. *Annual Review of Immunology*.

[8] Dejaco C, Duftner C, Grubeck-Loebenstein B, Schirmer M (2006). Imbalance of regulatory T cells in human autoimmune diseases. *Immunology*.

[9] Von Boehmer H (2005). Mechanisms of suppression by suppressor T cells. *Nature Immunology*.

[10] Collison LW, Workman CJ, Kuo TT (2007). The inhibitory cytokine IL-35 contributes to regulatory T-cell function. *Nature*.

[11] Ait-Oufella H, Salomon BL, Potteaux S (2006). Natural regulatory T cells control the development of atherosclerosis in mice. *Nature Medicine*.

[12] Bluestone JA, Tang Q (2005). How do CD4^+^CD25^+^ regulatory T cells control autoimmunity?. *Current Opinion in Immunology*.

[13] Jiang H, Chess L (2006). Regulation of immune responses by T cells. *The New England Journal of Medicine*.

[14] Möttönen M, Heikkinen J, Mustonen L, Isomäki P, Luukkainen R, Lassila O (2005). CD4^+^ CD25^+^ T cells with the phenotypic and functional characteristics of regulatory T cells are enriched in the synovial fluid of patients with rheumatoid arthritis. *Clinical and Experimental Immunology*.

[15] Lee SH, Goswami S, Grudo A (2007). Antielastin autoimmunity in tobacco smoking-induced emphysema. *Nature Medicine*.

[16] McGarvey LP, John M, Anderson JA, Zvarich M, Wise RA (2007). Ascertainment of cause-specific mortality in COPD: operations of the TORCH Clinical Endpoint Committee. *Thorax*.

[17] Bourbeau J, Christodoulopoulos P, Maltais F, Yamauchi Y, Olivenstein R, Hamid Q (2007). Effect of salmeterol/fluticasone propionate on airway inflammation in COPD: a randomised controlled trial. *Thorax*.

[18] Harita S, Kuyama S, Okada T, Tanizaki Y (2008). Effect of long-term and low-dose administration of erythromycin on proliferation of T lymphocytes stimulated with mitogens. *Journal of Chemotherapy*.

[19] Sun L, Liu J, Cui D (2010). Anti-inflammatory function of withangulatin a by targeted inhibiting COX-2 expression via MAPK and NF-*κ*B pathways. *Journal of Cellular Biochemistry*.

[20] Shinkai M, Foster GH, Rubin BK (2006). Macrolide antibiotics modulate ERK phosphorylation and IL-8 and GM-CSF production by human bronchial epithelial cells. *American Journal of Physiology*.

[21] Suzuki T, Yamaya M, Sekizawa K (2002). Erythromycin inhibits rhinovirus infection in cultured human tracheal epithelial cells. *American Journal of Respiratory and Critical Care Medicine*.

[22] Nagai H, Shishido H, Yoneda R, Yamaguchi E, Tamura A, Kurashima A (1991). Long-term low-dose administration of erythromycin to patients with diffuse panbronchiolitis. *Respiration*.

[23] Keicho N, Kudoh S (2002). Diffuse panbronchiolitis: role of macrolides in therapy. *American Journal of Respiratory Medicine*.

[24] Azuma A, Kudoh S (2006). Diffuse panbronchiolitis in East Asia. *Respirology*.

[25] King P (2007). Is there a role for inhaled corticosteroids and macrolide therapy in bronchiectasis?. *Drugs*.

[26] Simpson JL, Powell H, Boyle MJ, Scott RJ, Gibson PG (2008). Clarithromycin targets neutrophilic airway inflammation in refractory asthma. *American Journal of Respiratory and Critical Care Medicine*.

[27] Hahn DL (2009). Macrolide therapy in asthma: limited treatment, long-term improvement. *European Respiratory Journal*.

[28] Zhong XN, Bai J, Shi HZ, Wu C, Liang GR, Feng ZB (2003). An experimental study on airway inflammation and remodeling in a rat model of chronic bronchitis and emphysema. *Zhonghua Jie He He Hu Xi Za Zhi*.

[29] Sorsa M, Einisto P, Husgafvel-Pursiainen K (1985). Passive and active exposure to cigarette smoke in a smoking experiment. *Journal of Toxicology and Environmental Health*.

[30] Thurlbeck WM (1967). The internal surface area of nonemphysematous lungs. *American Review of Respiratory Disease*.

[31] Seemungal TAR, Wilkinson TMA, Hurst JR, Perera WR, Sapsford RJ, Wedzicha JA (2008). Long-term erythromycin therapy is associated with decreased chronic obstructive pulmonary disease exacerbations. *American Journal of Respiratory and Critical Care Medicine*.

[32] Rubin BK, Henke MO (2004). Immunomodulatory activity and effectiveness of macrolides in chronic airway disease. *Chest*.

[33] Nakanishi Y, Kobayashi D, Asano Y (2009). Clarithromycin prevents smoke-induced emphysema in mice. *American Journal of Respiratory and Critical Care Medicine*.

[34] Stefanska AM, Walsh PT (2009). Chronic obstructive pulmonary disease: evidence for an autoimmune component. *Cellular and Molecular Immunology*.

[35] Ishida Y, Abe Y, Harabuchi Y (2007). Effects of macrolides on antigen presentation and cytokine production by dendritic cells and T lymphocytes. *International Journal of Pediatric Otorhinolaryngology*.

[36] Ito T, Ito N, Hashizume H, Takigawa M (2009). Roxithromycin inhibits chemokine-induced chemotaxis of Th1 and Th2 cells but regulatory T cells. *Journal of Dermatological Science*.

[37] Yasutomi M, Ohshima Y, Omata N (2005). Erythromycin differentially inhibits lipopolysaccharide- or poly(I:C)-induced but not peptidoglycan-induced activation of human monocyte-derived dendritic cells. *The Journal of Immunology*.

